# Evaluation of anti-viral activity of *Jatropha curcas* leaf extracts against potentially drug-resistant HIV isolates

**DOI:** 10.1186/1471-2334-12-S1-P14

**Published:** 2012-05-04

**Authors:** Ritwik Dahake, Soumen Roy, Deepak Patil, Abhay Chowdhary, Ranjana A Deshmukh

**Affiliations:** 1 Department of Virology, Haffkine Institute, Mumbai 400012, Maharashtra, India

## Background

Drug-resistant HIV, a major global concern, warrants the development of novel anti-virals as alternative and inexpensive therapy. In the current study, we isolated potentially drug-resistant HIV and assessed previously unreported anti-viral activity of *Jatropha curcas* leaf extracts.

## Methods

*In vitro* micro-co-culture was employed for virus isolation followed by drug susceptibility assays to determine resistance to Azidothymidine (AZT) and Lamivudine (3TC).

*Jatropha curcas*leaves were extracted using Soxhlet apparatus. Methanolic (ME) and aqueous (AE) extracts were chosen for further study. Secondary metabolites were detected by High-Performance Thin Laye rChromatography and *in vitro* cytotoxicity established by MTT assay. Anti-viral activity was evaluated by p24 inhibitionin post- and pre-infection interaction studies.

## Results

Seven HIV isolates were obtained (isolation rate: 23.33%) with drug IC_50_ values ranging from 0.001418-82.73 µM AZT and 2.645-15.35 µM 3TC.

Tannins, flavonoids, saponins were detected in AE and flavonoids, saponins in ME while CC_50_ values were 32.07 mg/mL AE and 35.5 mg/mL ME.

In post-infection studies (4 isolates), IC_50_ values were ranging from 0.0255-0.4137 mg/mL AE and 0.00073-0.1278 mg/mL ME; pre-infection studies (1 isolate) showed 100% p24 inhibition by ME and 97.19% p24 inhibition by AE at 25 mg/mL each.

## Conclusion

HIV isolates potentially resistant to AZT/3TC were obtained; genotypic drug resistance is being ascertained. *Jatropha curcas* leaf extracts showed effective anti-viral and probable entry inhibition activity against potentially drug-resistant HIV, which has not been reported earlier. We conclude that *Jatropha curcas*is a good candidate for anti-HIV therapy with further research.

